# A Rare Differential for Myalgia and Fever Associated With Cervical and Axillary Lymphadenopathy Presenting via Same Day Emergency Care

**DOI:** 10.7759/cureus.96947

**Published:** 2025-11-16

**Authors:** Robert Baxter, Corinne Russell, Katharine Benedict

**Affiliations:** 1 Internal Medicine, Lister Hospital, Stevenage, GBR

**Keywords:** autoimmune, differential diagnosis, kikuchi-fujimoto disease, lymphoma, necrotizing lymphadenitis

## Abstract

Kikuchi-Fujimoto disease (KFD) is a rare, self-limiting necrotising lymphadenitis, mainly affecting young adults, and commonly presenting with fevers and lymphadenopathy. Diagnosis is confirmed by histopathological examination via lymph node biopsy, and management is primarily supportive with non-steroidal anti-inflammatory drugs and/or a tapering course of steroids.

We herein describe a case of a 23-year-old Caucasian female with severe KFD. She presented to the same day emergency care (SDEC) with a six-week history of systemic symptoms, including myalgia, arthralgia, fevers, and weight loss. Her initial blood tests showed normocytic anaemia, leukopenia, abnormal liver function tests, and a raised erythrocyte sedimentation rate. Notable aspects of this case include the large range of presenting symptoms, a coexisting Epstein-Barr virus (EBV) infection, and a delay in diagnosis of 14 days with a long inpatient admission.

KFD poses a diagnostic dilemma due to its non-specific presentation, with differentials from a range of varying aetiologies, including infective (EBV), rheumatological (systemic lupus erythematosus (SLE), adult Still's disease), haematological (lymphoma), and others (sarcoidosis, tuberculosis). For our patient, the early working diagnoses of SLE and lymphoma prompted consideration of a transfer to a specialist haematology oncology bed.

This case highlights the importance of early histopathological diagnoses in KFD and the need for a multidisciplinary approach, including haematology colleagues, commenced early in the acute setting to avoid misdiagnoses and unnecessary anxiety for patients. Currently, no specific national guidelines exist for KFD, and management is focused on supportive care. By sharing our experience, we hope to increase awareness of KFD's clinical presentation, diagnosis, and management amongst clinicians to avoid diagnostic delay or unnecessary interventions and guide timely therapeutic decisions.

## Introduction

Kikuchi-Fujimoto disease (KFD) is a rare and under-recognised form of necrotising lymphadenitis. KFD has a nonspecific clinical presentation of fever, cervical lymphadenopathy, and systemic symptoms, including night sweats, weight loss, and myalgia. The first cases of KFD worldwide were identified in Japan in 1972 by the pathologists Kikuchi and Fujimoto [1]. Most reported cases of KFD have been in Asia, with very few cases reported in Europe, and the prevalence in the United Kingdom remains unknown [2,3].

Whilst the aetiology of KFD remains unclear, there are several hypotheses suggesting a viral or autoimmune trigger. KFD is most prevalent in young adults, with most reported cases being in those younger than 30 years old. Although previously thought to be more prevalent in young women, recent literature suggests a less pronounced gender disparity [4].

Given the similarities in presentation between KFD and several other causes of lymphadenopathy, such as systemic lupus erythematosus (SLE), lymphoma, or infectious diseases, KFD is difficult to diagnose from signs and symptoms alone. The diagnosis of KFD is confirmed through histopathological evaluation of an excisional lymph node biopsy. Characteristic findings include focal areas of necrosis within the lymph nodes, accompanied by a high presence of histiocytes and karyorrhexis. Notably, there is a distinct absence of neutrophils [4].

The inflammatory cascade that occurs in KFD and resultant activation of T lymphocytes and histiocytes results in tissue necrosis within lymph nodes and the release of inflammatory cytokines. There are three histological phases: ​(1) the proliferative phase, seen in the early-mid stages of the disease. The proliferative phase is characterised by follicular hyperplasia with histiocyte and lymphocyte infiltration and an absence of neutrophils. (2) The necrotising phase. This stage signifies the peak of the disease, with extensive necrotic foci and histiocyte karyorrhectic debris (fragmentation of nuclear material). There remains an absence of neutrophils, helping to distinguish KFD from an infectious diagnosis. (3) The xanthomatous phase, seen in the late phases of the disease. There is regression of the necrosis with infiltration of foamy histiocytes [4].

KFD is a self-limiting disease with treatment typically given for symptom control. Management of KFD predominantly involves analgesics such as non-steroidal anti-inflammatories to treat pain, alongside antipyretics to treat fever. The symptoms usually resolve within weeks to months, and management is therefore supportive. However, in patients with severe or persistent symptoms, corticosteroids may be used [4].

KFD remains a diagnostic challenge for clinicians, and early histopathological evaluation is crucial to allow physicians to distinguish it from other conditions with similar presentations, such as lymphoma and SLE, and confirm the diagnosis.

## Case presentation

We present the case of a 23-year-old Caucasian female seen in the same day emergency care (SDEC) unit with a six-week history of multiple systemic symptoms, including myalgia, arthralgia, persistent fever, weight loss (5 kg), and a papular rash on her hands (starting on the palms and spreading to the back). She was referred by her general practitioner (GP) for deranged liver function tests (LFTs) with anaemia. Her symptoms had progressively worsened over a six-week period, against a background of feeling generally unwell for one year.

She had a past medical history of Hashimoto’s thyroiditis and was on levothyroxine (100-150 micrograms). There was a family history of rheumatoid arthritis, but no other family history of note. There was no history of foreign travel, intravenous drug use, insect bites, smoking, or excessive alcohol consumption.

She reported a history of feeling unwell for up to a year, which she had attributed to deranged thyroid function results requiring alterations to her levothyroxine dose. She was unable to continue with her university course and withdrew due to ongoing illness.

Examination findings revealed widespread cervical lymphadenopathy, a papular rash on the palmar surface of the left hand, and generalised abdominal tenderness.

Initially, she was referred to the hepatology, rheumatology, and haematology teams.

Timeline of events

**Figure 1:**
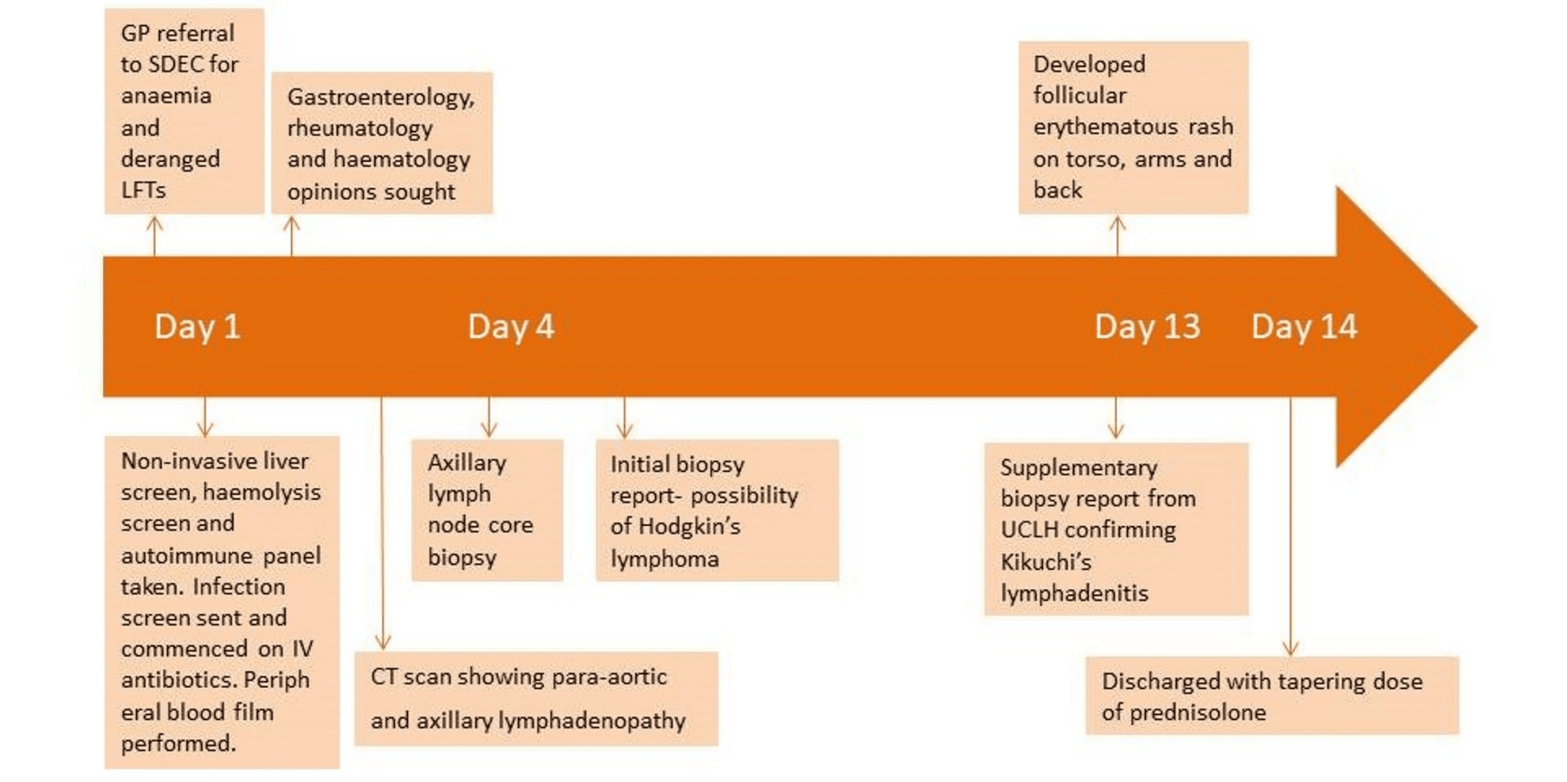
Timeline of events during admission. GP: general practitioner; SDEC: same day emergency care; LFT: liver function test; UCLH: University College London Hospitals.

The timeline of events is presented in Figure 1.

Investigations

Initial blood tests demonstrated a normocytic anaemia, neutropenia, lymphopenia, abnormal LFTs, and a raised erythrocyte sedimentation rate, as shown in Table 1. Early working differential diagnoses included autoimmune/viral hepatitis, Wilson’s disease, SLE, and lymphoma. A full non-invasive liver screen, blood film, and CT of the chest, abdomen, and pelvis were requested.

**Table 1:** Laboratory investigations. GP: general practitioner; ELISA: enzyme-linked immunosorbent assay; EBV: Epstein-Barr virus; VCA: viral capsid antigens; CMV: cytomegalovirus.

Investigations		Reference range	Dates		
			08/04/2024 - GP bloods		Admission bloods
Full blood count	White cell count	4-11 × 10^9^/L	2.6		2.3
	Neutrophil count	2-8 × 10^9^/L	1.70		1.54
	Lymphocyte count	1-4.5 × 10^9^/L	0.63		0.62
	Platelets	150-400 × 10^9^/L	323		272
	Haemoglobin	115-160 g/L	85		74
Liver function tests	Alanine transferase	7-40 U/l	463		288
	Alkaline phosphatase	30-130 U/l	297		306
	Gamma-glutamyl transferase	0-37 U/l	278		295
	Bilirubin	0-21 umol/l	16		9
Erythrocyte sedimentation rate		1-19 mm/hour	99		94
C-reactive protein		0-6 mg/l	5		4.7
Amylase		30-118 U/l			119
Creatinine		45-84 umol/L	59		
Ferritin		13-150 ug/L	2738.4		4337
Autoantibodies	Anti-nuclear antibody (HEP2)				Positive homogeneous pattern
	ANA ELISA (ENA, DSDNA and centromere)	0.0-0.9 units			1.3
	DNA (DS) antibody ELISA	0-10 iu/ml			
	Extractable nuclear antigens (ENA): RNP, SM, RO, LA, SCL, JO1, & CE	0-0.9 ratio			0.5
Thyroid function tests	Thyroid-stimulating hormone	0.27-4.2 mU/L	9.26		4.77
	T4	11-22 pmol/L	22.1		24.8
Non-invasive liver screen	EBV VCA IgM + IgG				Detected
	Leptospira IgM				Not detected
	HIV antigen/antibody				Negative
	CMV IgG				Not detected
	Hepatitis screen				Hepatitis not detected
	Mitochondrial antibody				Negative
	Smooth muscle negative				Negative
	Liver-kidney microsomal antibody				Negative
	Gastric parietal cell antibody				Negative
	Anti-HBC (Centaur) panel				Not detected
	Copper	12-20.2 umol/l			20.8
	Caeruloplasmin	0.2-0.4 g/l			0.36
Immunoglobulins	IgG	6.00-16.00 g/L			21.82
	IgA	0.80-2.80 g/L			4.13
	IgM	0.50-1.90 g/L			2.16
Complement	C3	0.75-1.65 g/l			1.03
	C4	0.14-0.54 g/l			0.29
Rheumatoid factor		0-13 iu/ml			<10
Lactate dehydrogenase		135-225 U/l			1146
Haptoglobin		0.4-2.8 g/L			<0.1

Sputum culture, viral swabs, and blood cultures were all negative. She had negative HIV, hepatitis A, hepatitis B, cytomegalovirus (CMV), and leptospira serology. Epstein-Barr virus (EBV) Epstein-Barr nuclear antigen (EBNA) IgG, EBV viral capsid antigen (VCA) IgM, and EBV DNA were detected.

Blood film report on 18/04/25 showed the following findings: RBC showed mild anisocytosis, normochromic with some macrocytes. WBC were mildly decreased in number, with some lymphocytes activated and with monocytes of large dimensions with polylobulated nucleus and mild basophilic cytoplasm with normal nuclear-cytoplasmic (N/C) ratio.

**Figure 2:**
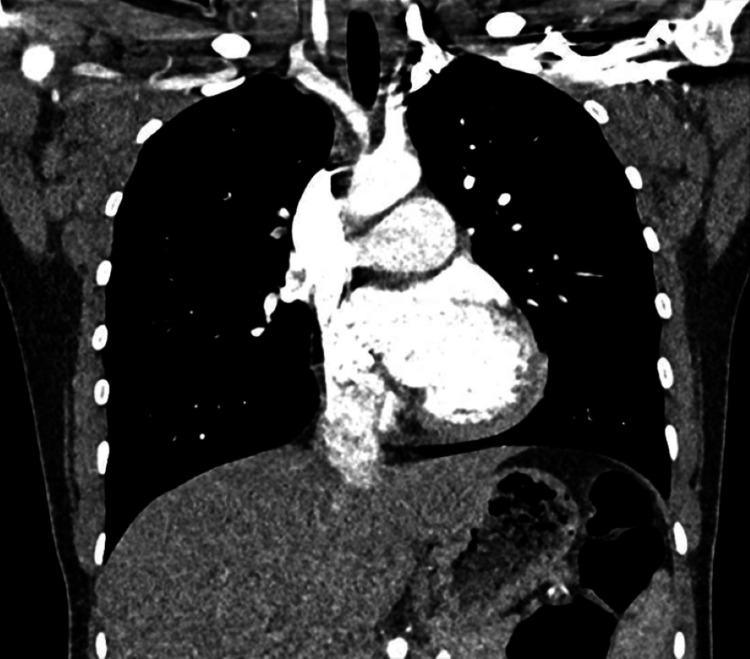
Coronal CT of the chest. A coronal contrast CT of the thorax demonstrating bilateral axillary lymphadenopathy in a patient later confirmed to have Kikuchi-Fujimoto disease. The enlarged lymph nodes are well-defined, discrete, and non-necrotic in appearance, with no mediastinal or hilar involvement.

**Figure 3:**
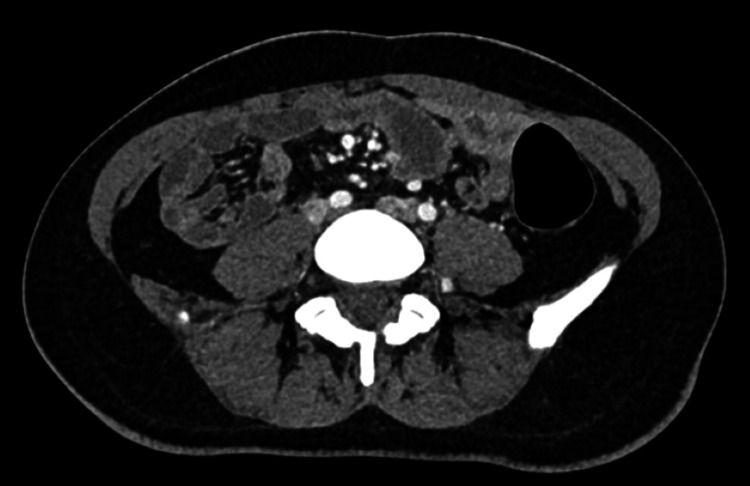
Axial CT of the abdomen. An axial contrast CT of the abdomen showing several mildly enlarged lymph nodes in the para-aortic region, adjacent to the abdominal aorta. The nodes are discrete and homogeneous in attenuation. There is no evidence of calcification or surrounding inflammatory change. These findings are suggestive of non-specific reactive lymphadenopathy.

CT scan report on 11/4/25 showed the following findings: bulky lymphadenopathy in both axillaries, left side worse, with further enlarged lymph nodes in the retroperitoneum. Appearances were suspicious for lymphoma (Figures 2, 3).

The case was discussed with the haematology team, who advised doing a lymph node biopsy and potentially a bone marrow biopsy. A blood film showed no signs of acute leukaemia but signs of cold agglutinin. Initial plans were made to transfer her to a specialist haematology-oncology bed.

Initial biopsy report on 12/04/24 was as follows: some of the large histiocytic cells were reminiscent of Hodgkin's cells, which were positive for CD30 with concurrent epithelial membrane antigen (EMA) staining. Very occasional classical-type Hodgkin's cells were noted. Some of these larger cells were positive for CD20 as well. A possibility of Hodgkin's lymphoma needed to be ruled out here. The case was referred to University College London Hospitals (UCLH) for further assessment.

**Figure 4:**
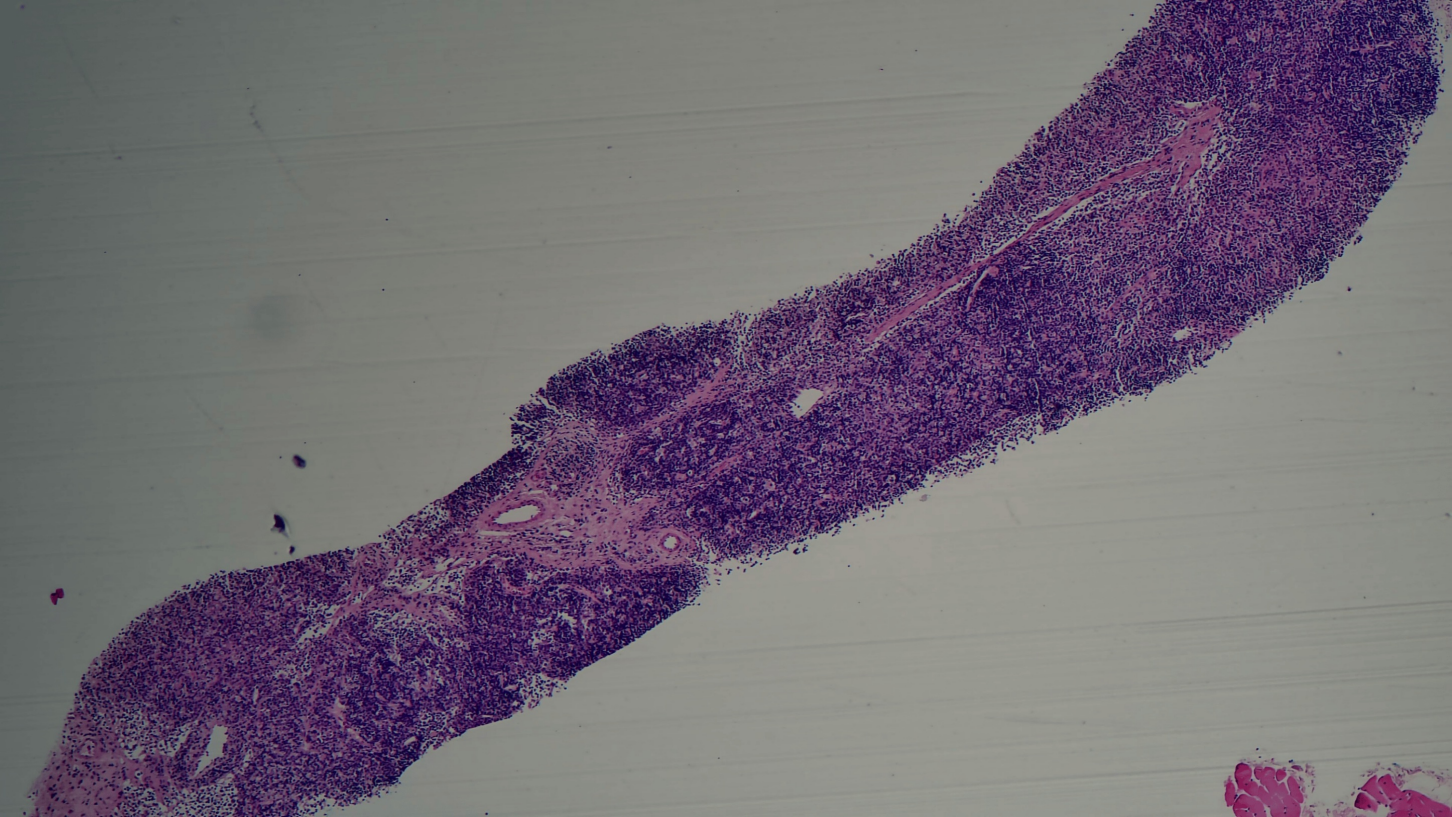
Lymph node biopsy micrographs (x10 magnification). Low-power view (×10) of the lymph node core biopsy showing pale-staining areas, particularly in the lower left corner of the micrograph. Micrographs and biopsy report by Dr. Yasotha Thevacumar and Dr. Amali Albert.

**Figure 5:**
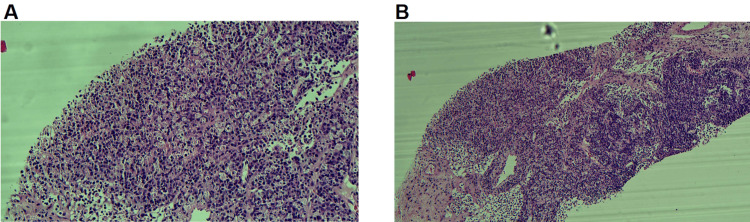
Lymph node biopsy micrographs: (A) x40 magnification and (B) x20 magnification. High-power views of the pale-staining area. (A) ×40 magnification and (B) ×20 magnification demonstrate histiocytes admixed with apoptotic bodies, consistent with histiocytic necrotising lymphadenitis, as seen in Kikuchi-Fujimoto disease. Micrographs and biopsy report by Dr. Yasotha Thevacumar and Dr. Amali Albert.

However, following her results being sent to UCLH for further assessment, a supplementary report received on 22/04/24 was as follows: ​​​​​​​small core biopsy of the lymph node showed patent sinuses, small mature lymphocytes, and focal collections of histiocytic cells with prominent apoptosis. CD3 and CD5 showed background T cells, and CD40 demonstrated loose collections of B cells. Scattered CD30-positive cells were present, but their appearances were not those of Hodgkin's disease. CD11c and CD68 confirmed collections of histiocytes. CD123 showed admixed plasmacytoid dendritic cells, and there was an associated population of CD8-positive T cells. Myeloperoxidase (MPO) showed granular cytoplasmic positivity in many of the histiocytes present. The appearances of biopsy findings were those of Kikuchi’s lymphadenitis. Lupus has been reported to lead to similar pathological changes, but in practice, this is very rare. There was no lymphoma. Left axillary lymph node core biopsy confirmed KFD (Figures 4, 5).

Treatment

Our patient was commenced on 40 mg prednisolone for one week, following a haematology review, with a plan to taper it by 5 mg weekly. She was also discharged with non-steroidal anti-inflammatory drugs (NSAIDs) and antihistamines after developing a new follicular erythematous rash on her upper arms, chest, abdomen, and back. It was unclear whether this rash was a drug-induced reaction or due to Kikuchi’s lymphadenitis. She was referred to dermatology and had ongoing follow-up arranged with haematology. She was discharged after a total 14-day admission. Ferritin and lactate dehydrogenase (LDH) levels were normalised post steroid treatment.

Follow-up

We followed up with our patient over a year later. She had since been diagnosed with Still's disease. Following her being discharged on oral steroids, she only showed mild improvement. She saw a private rheumatologist who performed a bone marrow biopsy (confirming no lymphoma) and subsequently diagnosed her with Still's disease. She was unable to distinguish whether her symptoms at that stage were a result of Kikuchi's lymphadenitis or Still's disease. She is currently on anakinra injections for her Still's disease and is doing well. She did report that she still gets flare-ups of lymphadenopathy, exactly how she initially presented to SDEC, which she feels is due to the Kikuchi lymphadenitis.

## Discussion

The aetiology of KFD remains debated, with no definitive cause identified. Several hypotheses have been proposed, including viral infections, autoimmune conditions, and genetic factors. Some researchers have suggested that viral infections may trigger KFD. Associations with viruses such as EBV, CMV, human herpesvirus 6 (HHV-6), and parvovirus B19 have been reported, with the viruses potentially resulting in immune dysregulation, as seen in KFD. Although EBV is the virus most commonly associated with KFD, no consistent viral agent has been definitively linked to KFD [5,6]. ​Interestingly, our patient tested positive for EBV serology, which could support the hypothesis that EBV could be a contributory factor in the development of KFD.

Bacterial agents, such as *Yersinia* or *Toxoplasma gondii*, have also been suggested, but no consistent findings across cases have confirmed this theory [4,6].

Another suggestion is that KFD may represent a self-limiting autoimmune process/condition. Several cases of KFD have been reported in patients with co-existing autoimmune conditions, commonly SLE, but also others such as rheumatoid arthritis, Sjogren's syndrome, Hashimoto's thyroiditis, systemic sclerosis, and antiphospholipid syndrome. KFD can precede, follow, or occur simultaneously with autoimmune disease [7,8]. In our case, the patient had a longstanding diagnosis of Hashimoto’s thyroiditis, and there was a family history of rheumatoid arthritis, supporting a potential link to an autoimmune trigger. Interestingly, she has since been diagnosed with Still’s disease. The coexistence of Hashimoto’s and Still’s disease supports the proposed association between autoimmune diseases and Kikuchi’s lymphadenitis.

There may also be a genetic predisposition in individuals diagnosed with KFD; however, research surrounding possible genetic factors is limited [9].

KFD can present with a wide array of symptoms, including fever, lymphadenopathy, arthralgia, fatigue, hepatosplenomegaly, and weight loss. The posterior cervical lymph nodes are most commonly affected; however, generalised lymphadenopathy may occur. In this patient, cervical, axillary, and retroperitoneal lymphadenopathy was noted. Cutaneous manifestations are also reported in patients with KFD, most often presenting as non-specific macules or papules. A transient papular rash was observed in this case, affecting the patient's palms and back [5,10].

Alongside the broad symptomatology of KFD, a wide range of laboratory abnormalities may be observed. Anaemia, leukopenia, elevated erythrocyte sedimentation rate, and raised LDH levels are common findings seen in KFD patients. Deranged LFTs may also occur. In this case, it is difficult to determine whether the hepatocellular dysfunction was due to the EBV infection, KFD itself, or a combination of both conditions [10].

KFD is frequently misdiagnosed, with lymphoma being a leading differential diagnosis in this case. In lymphoma patients, B symptoms (fever, night sweats, weight loss) commonly accompany lymphadenopathy and fatigue. As illustrated in this case, KFD can have a very similar clinical picture, with fever and lymphadenopathy as typical predominant symptoms. In this case, the patient exhibited fever, weight loss, fatigue and lymphadenopathy. Haematological findings further raised the clinical suspicion of lymphoma. Anaemia, thrombocytopenia, and leukopenia may indicate bone marrow involvement in lymphoma. In this case, the patient's initial blood work revealed leukopenia, anaemia, and an elevated LDH (1146 U/L), supporting the possibility of a haematological malignancy [11].

Histologically, the lymph node biopsy demonstrated CD30-positive cells, commonly seen in classic Hodgkin's lymphoma. In addition to this, occasional cells resembling Hodgkin/Reed-Sternberg cells were observed, making Hodgkin's lymphoma a reasonable possibility [12]. However, upon further review by the UCLH, they commented on "focal collections of histiocytic cells with prominent apoptosis" and "collections of histiocytes", features more consistent with KFD. Biopsy findings in KFD typically show areas of necrosis with infiltrate consisting of crescentic histiocytes, plasmacytoid dendritic cells, and lymphocytes. A hallmark finding in KFD is apoptosis, with an absence of neutrophils. There is also a predominance of CD8+ T-cells, and the lymphoid population is polyclonal with an absence of atypical lymphocyte cells [5,13]. A significant diagnostic challenge in cases of KFD is the timely differentiation from lymphoma, and it is these hallmark histopathological findings seen on biopsy that can confirm a diagnosis of KFD. Given that the initial secondary care biopsy report was unable to rule out lymphoma and a further 10-day delay occurred before the tertiary centre report was received, further histological education on the findings of KFD is required amongst clinicians to improve the efficiency and awareness of a KFD diagnosis.

In addition to lymphoma, the other differential diagnoses of infections (e.g., tuberculosis and EBV), sarcoidosis, and autoimmune diseases must also be excluded. SLE is another condition that KFD is commonly mistaken for. SLE is a multisystem autoimmune disease presenting with a variety of symptoms due to multiorgan involvement. Common symptoms include fever, fatigue, arthralgia, and weight loss. Many patients exhibit cutaneous manifestations, such as a malar rash, though this is not always present. In terms of laboratory findings, anaemia is often present alongside leukopenia. Thrombocytopenia may also be seen. The diagnosis of SLE can be challenging and is based on a combination of clinical features, autoantibody testing, and other laboratory findings. Whilst antinuclear antibodies are positive in nearly all cases of SLE, a positive antinuclear antibody (ANA) can also be seen in other conditions as well as in healthy individuals. In this case, the patient had slightly elevated ANA levels, but negative dsDNA and extractable nuclear antigen (ENA) levels and normal complement levels. Histologically, KFD and SLE are markedly similar. Studies have shown that in approximately 20% of cases of SLE, lymph node biopsies may be indistinguishable from KFD. However, most patients with SLE also have distinctive clinical features that are not typically seen in KFD, such as a butterfly rash, photosensitivity, and discoid lesions, in addition to the presence of autoantibodies (for example, anti-dsDNA and anti-Sm antibodies). This highlights the importance of testing for these autoantibodies early on in the work-up of patients presenting with features overlapping those of both KFD and SLE (Table 2) [14,15].

**Table 2:** Table highlighting the key differences between Kikuchi-Fujimoto disease, systemic lupus erythematosus, and lymphoma. Source [1,2,4,8,11-15]. ANA: antinuclear antibody; dsDNA: double-stranded DNA; ENA: extractable nuclear antigens; NSAIDs: non-steroidal anti-inflammatory drugs.

	Kikuchi-Fujimoto disease (KFD)	Systemic lupus erythematosus (SLE)	Lymphoma
Histopathology	Necrotising lymphadenitis with karyorrhectic debris, abundant apoptotic bodies, and absence of neutrophils.	Necrotising lymphadenitis can be similar to KFD, but usually with haematoxylin bodies, immune complex deposition, and sometimes neutrophils.	May show necrosis, but typically with neutrophilic infiltration. Reed-Sternberg cells in Hodgkin’s lymphoma.
Histology cell types	Crescentic histiocytes, plasmacytoid dendritic cells, and CD8+ T-cell predominance.	Polymorphous infiltrate: lymphocytes, plasma cells, histiocytes, occasional neutrophils.	Monomorphic population of malignant lymphoid cells (e.g., Reed-Sternberg cells in Hodgkin’s; atypical B or T cells in non-Hodgkin's).
Autoantibodies	ANA may be weakly positive; dsDNA and ENA are usually negative.	ANA is almost always positive; anti-dsDNA, anti-Smith and other ENA antibodies are positive in many patients; complement (C3, C4) levels are often low.	Typically negative, unless paraneoplastic features are present.
Immunophenotyping	CD68+, MPO+, CD123+ histiocytes; abundant CD8+ T-cells. Polyclonal pattern.	Polyclonal B and T lymphocytes, with immune complex deposition detectable by immunofluorescence.	Hodgkin cells: CD15+, CD30+ (in classical Hodgkin’s lymphoma); non-Hodgkin: variable markers depending on subtype. Often monoclonal.
Clinical features	Fever, cervical/axillary lymphadenopathy, rash, arthralgia, fatigue, mild hepatosplenomegaly. Usually self-limiting.	Multisystem disease: fever, malar rash, photosensitivity, arthralgia, nephritis, CNS involvement, cytopenias. Chronic, relapsing.	"B symptoms" (fever, night sweats, weight loss), painless lymphadenopathy, organomegaly. Can progress rapidly.
Treatment	Supportive (NSAIDs, steroids for severe cases). Self-resolving over weeks to months.	Immunosuppression (steroids, hydroxychloroquine, biologics). Lifelong monitoring.	Chemotherapy, radiotherapy, or targeted agents, depending on subtype.

Given the association between KFD and autoimmune diseases, particularly SLE, as well as the overlap in clinical features, we recommend structured follow-up for all patients diagnosed with KFD. This follow-up could include an initial review in primary care a couple of months post treatment to ensure symptom resolution, along with patient education on the early warning signs of autoimmune disease. Repeat autoantibody testing should also be considered during follow-up, with a low threshold for specialist referral and further investigation if new or persistent symptoms arise. In our case, the patient did not improve as expected with corticosteroid treatment and was subsequently diagnosed with Still’s disease. Retrospectively, it is possible that some of her initial symptoms were due to a coexistence of both Kikuchi’s lymphadenitis and Still’s disease. The diagnosis of Still’s so soon after KFD highlights the importance of close follow-up in these patients. As KFD is generally a self-limiting disease, a slower-than-expected response to NSAIDs or steroids should prompt clinicians to consider a concurrent diagnosis alongside KFD.

This case highlights the diagnostic uncertainty clinicians may face when presented with the diverse symptomatology of KFD. In this case, there was a 14-day delay in diagnosis, with the final biopsy report confirming KFD not received until 22/04/24. Prolonged inpatient admissions not only have a detrimental effect on patient well-being, contributing to anxiety and distress, but also place a considerable financial burden on the NHS. Multiple factors contributed to the delay in diagnosis seen in this case. The patient was initially admitted under gastroenterology due to deranged LFTs. This meant she was not regularly seen by the other specialities involved in her case, such as haematology, and a lot of time was spent trying to communicate between different specialities. Furthermore, the wait for the tertiary centre biopsy report further extended the diagnostic timeline. Limited awareness and recognition of KFD among clinicians are key contributors to such diagnostic delays. During the entire admission, KFD was not considered as a potential diagnosis by any members of the multi-disciplinary teams involved, highlighting the importance of wider education on the clinical presentation of KFD. In diagnostically complex cases, such as these, maintaining a wide differential diagnosis is paramount to avoid missing rarer conditions like KFD.

This case is unique, given the rarity of KFD cases reported in the UK and the limited literature describing its presentation in Caucasian patients, given that it is predominantly seen in Asian populations. The majority of KFD cases in the UK and Europe described in the current literature occur predominantly in Asian women in their third decade of life [2]. Furthermore, reported cases of KFD in the UK and Europe rarely include follow-up of patients a year after their initial KFD diagnosis. In our case, this follow-up revealed a rare co-occurrence of KFD and Still’s disease, highlighting the diagnostic challenges such overlapping presentations can pose in clinical practice.

There is a clear gap in the clinical guidance for KFD management. Further research is needed to develop guidelines for the management of KFD and to improve clinician awareness and understanding of how KFD presents, especially in Europe and America, where it is less well-recognised.

## Conclusions

Early diagnosis of KFD remains a challenge, given its clinical similarity to other conditions, particularly lymphoma and SLE. Although KFD is a benign and self-limiting disease, accurate and timely diagnosis is essential to avoid unnecessary treatment and interventions, reduce prolonged diagnostic uncertainty, alleviate patient distress, and minimise healthcare costs. Clinicians should be aware of the presentation of KFD, and in cases where it may be a differential diagnosis, prompt lymph node biopsy is recommended to confirm the diagnosis.
